# Regulation of phase separation and antiviral activity of Cactin by glycolytic enzyme PGK via phosphorylation in *Drosophila*

**DOI:** 10.1128/mbio.01378-23

**Published:** 2024-03-06

**Authors:** Dongchao Chen, Chang Shi, Wen Xu, Qiqi Rong, Qingfa Wu

**Affiliations:** 1 Department of Pharmacy, The First Affiliated Hospital of USTC, Division of Life Sciences and Medicine, University of Science and Technology of China, Hefei, Anhui, China; 2 Division of Molecular Medicine, CAS Key Laboratory of Innate Immunity and Chronic Disease, University of Science and Technology of China, Hefei, Anhui, China; 3 Anhui Provincial Key Laboratory of Precision Pharmaceutical Preparations and Clinical Pharmacy, Hefei, Anhui, China; University of Pennsylvania, Philadelphia, Pennsylvania, USA; Johns Hopkins University, Baltimore, USA

**Keywords:** Cactin, antiviral immunity, liquid–liquid phase separation, phosphoglycerate kinase, *Drosophila* C virus, *Drosophila melanogaster*

## Abstract

Liquid–liquid phase separation (LLPS) plays a crucial role in various biological processes in eukaryotic organisms, including immune responses in mammals. However, the specific function of LLPS in immune responses in *Drosophila melanogaster* remains poorly understood. Cactin, a highly conserved protein in eukaryotes, is involved in a non-canonical signaling pathway associated with Nuclear factor-κB (NF-κB)-related pathways in *Drosophila*. In this study, we investigated the role of Cactin in LLPS and its implications for immune response modulation. We discovered that Cactin undergoes LLPS, forming droplet-like particles, primarily mediated by its intrinsically disordered region (IDR). Utilizing immunoprecipitation and mass spectrometry analysis, we identified two phosphorylation sites at serine residues 99 and 104 within the IDR1 domain of Cactin. Co-immunoprecipitation and mass spectrometry further revealed phosphoglycerate kinase (PGK) as a Cactin-interacting protein responsible for regulating its phosphorylation. Phosphorylation of Cactin by PGK induced a transition from stable aggregates to dynamic liquid droplets, enhancing its ability to interact with other components in the cellular environment. Overexpression of PGK inhibited *Drosophila* C virus (DCV) replication, while PGK knockdown increased replication. DCV infection also increased Cactin phosphorylation. We also found that phosphorylation enhances the antiviral ability of Cactin by promoting liquid-phase droplet formation. These findings demonstrate the role of Cactin-phase separation in regulating DCV replication and highlight the modulation of its antiviral function through phosphorylation, providing insights into the interplay between LLPS and antiviral defense mechanisms.

**IMPORTANCE:**

Liquid–liquid phase separation (LLPS) plays an integral role in various biological processes in eukaryotic organisms. Although several studies have highlighted its crucial role in modulating immune responses in mammals, its function in immune responses in *Drosophila melanogaster* remains poorly understood. Our study investigated the role of Cactin in LLPS and its implications for immune response modulation. We identified that phosphoglycerate kinase (PGK), an essential enzyme in the glycolytic pathway, phosphorylates Cactin, facilitating its transition from a relatively stable aggregated state to a more dynamic liquid droplet phase during the phase separation process. This transformation allows Cactin to rapidly interact with other cellular components, enhancing its antiviral properties and ultimately inhibiting virus replication. These findings expand our understanding of the role of LLPS in the antiviral defense mechanism, shedding light on the intricate mechanisms underlying immune responses in *D. melanogaster*.

## INTRODUCTION

Liquid–liquid phase separation (LLPS) is a process that occurs in cells where certain proteins and other biomolecules separate from the surrounding solution to form distinct liquid-like compartments (1). These compartments, known as membraneless organelles or biomolecular condensates, are dynamic structures that play important roles in various cellular functions (2–5). Several membraneless organelles are formed through LLPS, including P bodies, stress granules, Cajal bodies, and nucleoli (3, 6–10). Biomolecular condensates can undergo a phase transition and transform into gel-like condensates that exhibit viscoelastic properties. This physical transformation creates a barrier that restricts the exchange of molecules with the external environment and inhibits the activity of separated proteins (5, 11, 12). Soluble proteins that possess modular structural domains, or intrinsically disordered regions (IDRs), are typically involved in the formation of biomolecular condensates through LLPS. These domains provide weak, multivalent interactions between molecules (8, 13, 14).

LLPS is driven by the physical properties of proteins and nucleic acids, such as their concentration, interactions, and post-translational modifications (2, 15, 16). When these molecules reach a critical concentration or undergo specific modifications, they can phase separate and form condensed droplets within the cell. These droplets are often highly dynamic and can rapidly assemble, disassemble, and fuse together, allowing for the exchange of molecules and information (17). For example, LET-36, an ortholog of the mammalian mTOR protein in *Caenorhabditis elegans*, can phosphorylate PGL-1 and promote the phase separation process (18). Protein kinases CK2 and S6K1 can phosphorylate the IDR domain of the FMRP protein, changing the charge or other properties of the IDR domain and promoting LLPS formation through multivalent interactions (19). During the asymmetric division of *Drosophila* neural stem cells, the Numb/Pon complex undergoes LLPS, which mediates polar enrichment of Numb in the bottom cortex of neural stem cells and regulates neural stem cell differentiation (20). Moreover, host cell proteins and nucleic acid molecules can also regulate innate immune responses through LLPS (21). Excessively produced 2'3'-cyclic GMP-AMP (cGAMP) triggers the phase separation of Endoplasmic reticulum (ER)-associated STING proteins in DNA virus-infected cells, preventing the overactivation of innate immunity (21). Although the role of LLPS in virus–host interactions has gained much attention, further research is necessary to comprehend how LLPS regulates virus replication.

PGK is an essential enzyme in the glycolytic pathway, catalyzing the conversion of 1,3-bisphosphoglycerate to 3-phosphoglycerate while generating ATP (22). Recent studies have discovered the various roles of PGK in viral infections, highlighting its importance in host–virus interactions (23–25). In the white leg shrimp (*Litopenaeus vannamei*), PGK has been found to play a pivotal role in the infection of the white spot syndrome virus (WSSV), a highly contagious and lethal virus in crustaceans (23). Knockdown of *LvPGK* has been shown to result in reduced WSSV replication and decreased mortality in *L. vannamei* (23). Similarly, PGK1, an isoform of PGK, has been demonstrated to facilitate the assembly of viral replicase complexes, a critical step in viral replication (24). Sendai virus, a negative-strand RNA virus, co-opts Pgk1 to promote viral mRNA synthesis, even though its enzymatic activity is not required for the stimulation of RNA synthesis (26). Moreover, in *Arabidopsis*, partial/recessive resistance to potyviruses is due to a single amino acid mutation in the conserved N-terminal portion of the chloroplast phosphoglycerate kinase (cPGK2), which is a nuclear gene encoding cPGK2 targeted to the chloroplast (27, 28). Furthermore, cPGK1 was found to bind to the 3′UTR of viral RNA and is involved in the localization of potexvirus RNA to chloroplasts, which is important for virus accumulation (25, 29). In *Nicotiana benthamiana*, mislocalized NbPGK1 can lead to a dominant negative effect on the coat protein accumulation of *BaMV* (25).

*Drosophila melanogaster* is a widely used model organism to investigate innate immunity (30–34). The conserved Toll and Imd pathways, which are counterparts of the mammalian NF-κB pathways, play crucial roles in the defense against bacterial, fungal, and viral infections in *Drosophila* (35–39). Recently, a non-canonical immune signaling pathway has been identified in *Drosophila*, in which the Cactin protein interacts with the transcription factors Deaf1 and RNA polymerase II to mediate the expression of antimicrobial peptides, instead of the NF-κB-type transcription factors Dorsal, Dif, and Relish (40). Cactin is a highly conserved protein in eukaryotes (41), which includes a nuclear localization signal (NLS), a coiled-coil (CC) domain, and a region with relatively low complexity that is rich in arginine and serine residues (42). The role of Cactin in NF-κB pathways and the induction of immune response genes has also been explored in other organisms (43, 44). Notably, Cactin restricts *Drosophila* C virus (DCV) replication in *D. melanogaster*, while Deaf1 knockdown has no significant effect on DCV replication, suggesting that the antiviral mechanism mediated by Cactin might be independent of the non-canonical Cactin-Deaf1 signaling pathway (40).

In this study, we made the novel discovery that Cactin undergoes phase separation. Specifically, we found that after DCV infection of S2 cells, PGK phosphorylates Cactin and regulates its phase separation process. This phosphorylation event leads to the transition of Cactin from stable aggregates to more dynamic liquid droplets, which can readily interact with other components in the cellular environment. Our data strongly suggest that the phase separation of Cactin is crucial for its antiviral function, given that this process effectively inhibits DCV replication.

## RESULTS

### Cactin forms droplet-like particles through LLPS in *Drosophila* cells

**Fig 1:**
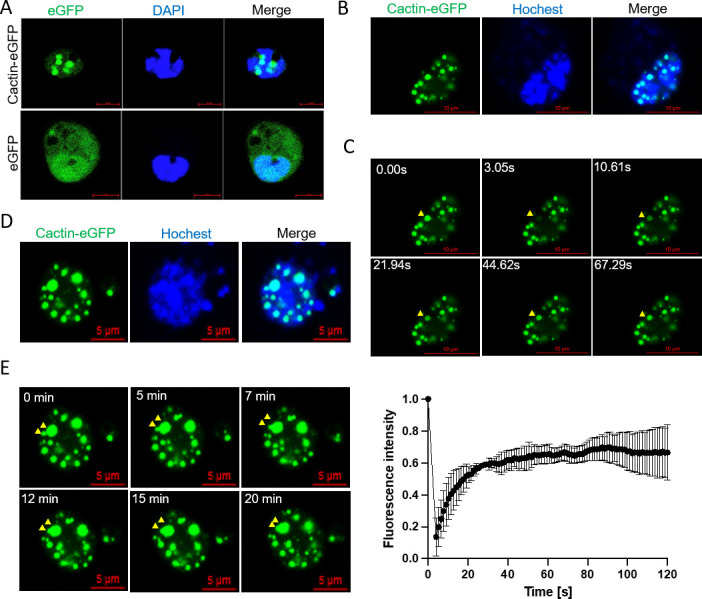
Cactin forms droplet-like particles through LLPS in *Drosophila* S2 cells (**A**) *Drosophila* S2 cells transfected with pMT-Cactin-eGFP or pMT-eGFP plasmids were stained with DAPI to visualize the nucleus. Confocal images depict the formation of Cactin-eGFP droplet-like structures, while free eGFP fails to form droplets (scale bar, 5 µm). (**B**) *Drosophila* S2 cells transfected with the pMT-Cactin-eGFP plasmid were stained with Hoechst 33342 to label the nucleus in living cells (scale bar, 10 µm). (**C**) FRAP analysis of Cactin droplet-like structures in S2 cells (same cell as panel B). Montages on the top illustrate the FRAP process of a droplet (scale bar, 10 µm). The position is indicated by a yellow arrow. The graph on the bottom displays the recovery of fluorescence intensity over time. Data correspond to mean ± SD with *n* = 3. (**D**) *Drosophila* S2 cells transfected with the pMT-Cactin-eGFP plasmid were stained with Hochest 33342 to label the nucleus in living cells (scale bar, 5 µm). (**E**) Representative images demonstrate the time-dependent fusion of two Cactin-eGFP droplets in S2 cells (same cell as panel D). Montages depict the phase fusion process of a droplet (scale bar, 5 µm). The position is indicated by a yellow arrow.

A common method for studying LLPS is to create fluorescent protein fusion constructs (45). To investigate the behavior of Cactin in cells, we transfected *Drosophila* S2 cells with either a pMT-Cactin-eGFP plasmid or a pMT-eGFP plasmid (control) and observed the fluorescence of Cactin-eGFP and eGFP using a laser confocal microscope. We observed droplet-like Cactin aggregates in the nucleus, which formed and remained localized (Fig. 1A). We aimed to clarify whether the Cactin-eGFP aggregates observed in the nucleus are concentrated liquid-phase droplets or solid-phase aggregates (46). To address this question, we employed fluorescence recovery after photobleaching (FRAP) to assess the mobility of Cactin-eGFP within punctate aggregates. We found that Cactin-eGFP forms multiple punctate nuclear aggregates that rapidly move from the surrounding nuclear regions to the bleached area upon photobleaching (Fig. 1B and C). We further used FRAP to quantify the molecular dynamics within the Cactin-eGFP droplets and found that approximately 60% of the Cactin-eGFP signal in the droplets gradually recovered within 120 s after photobleaching (Fig. 1C). The recovery of fluorescence within the condensate indicates that Cactin-eGFP forms concentrated liquid-phase droplets rather than solid-phase aggregates.

Additionally, we investigated the occurrence of fusion events within phase-separated Cactin-eGFP structures, which exhibit properties akin to liquid droplets. Through confocal fluorescence microscopy, we observed that exogenously expressed Cactin-eGFP droplets initially formed small aggregates, gradually merging to create larger droplets over time (Fig. 1D and E). These observations strongly suggest that the Cactin protein undergoes LLPS within living cells, giving rise to droplet-like structures that display typical liquid behaviors such as fusion and flow. Collectively, our findings provide compelling evidence that the *Drosophila* Cactin protein is capable of undergoing phase separation in the cellular context.

### The IDR1 domain of Cactin is the critical region affecting phase separation

**Fig 2:**
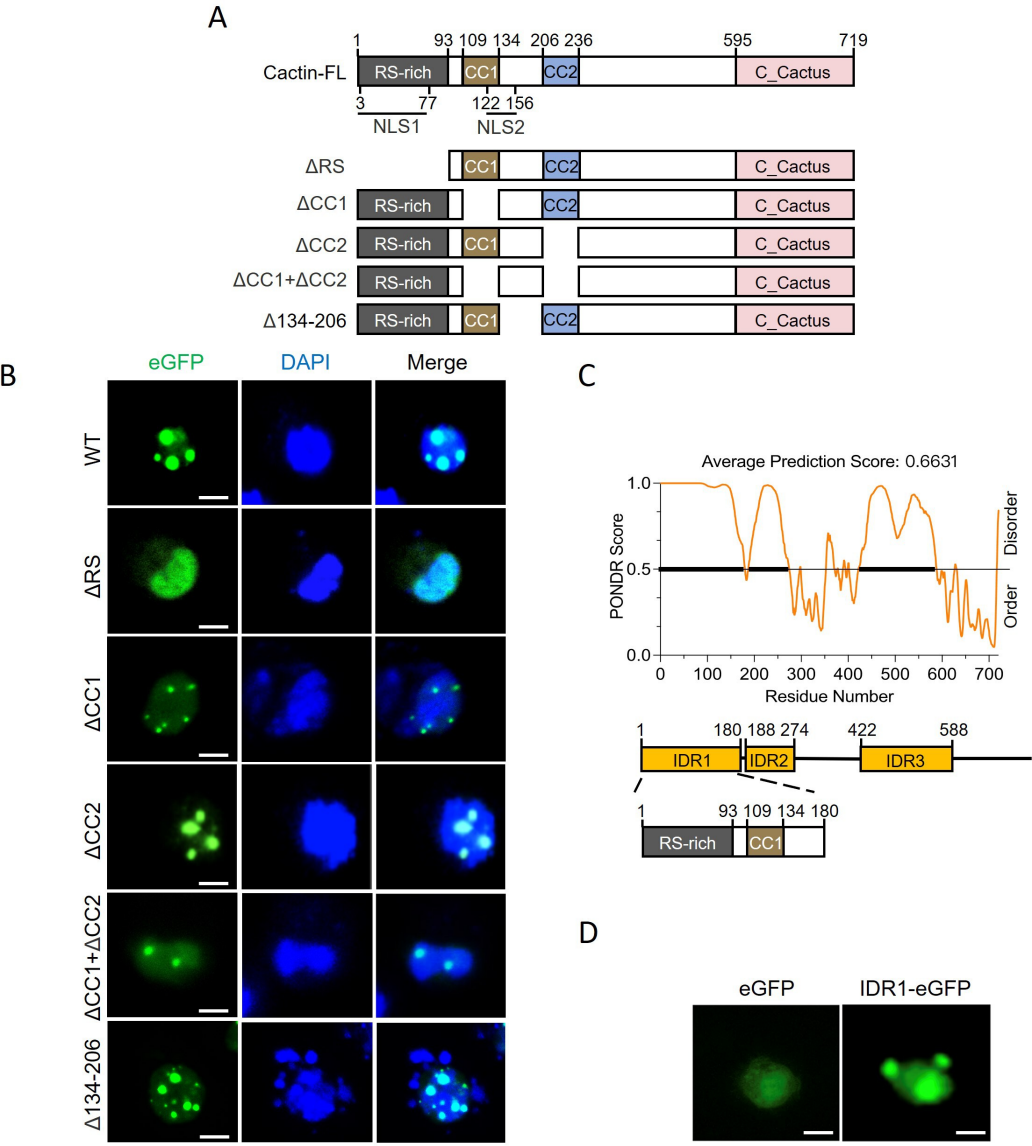
The IDR1 domain of Cactin is crucial for phase separation. (**A**) Domain map of Cactin, featuring an N-terminal RS-rich domain, two coiled coils (CC1 and CC2), and the Cactus-interacting domain at the C-terminus. Schematic representation of the two NLS domains of Cactin, with positions of the five deletion variants indicated. (**B**) Confocal images of S2 cells transfected with Cactin-eGFP WT or variants, treated with DAPI for nuclear visualization (scale bar, 5 µm). (**C**) Disorder confidence score (top), diagrammatic representation of identified Cactin IDR domains (middle), and the structural domain encompassed by IDR1 (bottom). IDRs were predicted using the online tool PONDR. (**D**) Confocal images illustrating the condensed state of eGFP and IDR1-eGFP in S2 cells (scale bars, 5 µm).

The *Drosophila* Cactin protein consists of an N-terminal arginine/serine-rich (RS-rich) domain (amino acids 1–93), two CC domains, CC1 (amino acids 109–134) and CC2 (amino acids 206–236), and a C-terminal conserved domain that interacts with Cactus (Fig. 2A). The NLS of Cactin, predicted by NLStradamus (47), are located at amino acids 3–77 within the RS-rich domain and amino acids 122–156, partially overlapping with the CC1 domain (Fig. 2A). To determine which domains are essential for the phase separation of Cactin, we generated ΔRS, ΔCC1+ΔCC2, ΔCC1, ΔCC2, and Δ134–206 mutant expression vectors that lack the RS-rich domain, both CC domains, CC1 domain and CC2 domain, and the region between CC1 and CC2, respectively (Fig. 2A). Fluorescence microscopy analysis of Cactin-eGFP WT and its mutants revealed that ΔRS inhibited phase separation, while ΔCC2, Δ134–206, and Cactin WT formed larger droplets, and ΔCC1 and ΔCC1+ΔCC2 formed smaller droplets (Fig. 2B). These findings suggest that the RS-rich domain is the critical region for regulating the phase separation of Cactin, while the CC1 domain also plays a partial role.

Given that protein IDRs are usually involved in phase separation (48), we used PONDR software to analyze the IDRs in *Drosophila* Cactin and identified three IDRs: IDR1 (amino acids 1–180), IDR2 (amino acids 188–274), and IDR3 (amino acids 422–588) (Fig. 2C). Notably, IDR1 encompasses the RS-rich and CC1 regions, which we already know to have a role in regulating the phase separation of Cactin. To confirm the effect of IDR1 on phase separation, we constructed an expression vector in which the IDR1 domain was fused to the N-terminus of eGFP. Fluorescence microscopy revealed that IDR1-eGFP formed droplets in cells, while control eGFP was diffusely distributed (Fig. 2D), providing further evidence that the IDR1 domain is the main region affecting phase separation of Cactin.

### IDR1 domain phosphorylation transitions Cactin from stable aggregates to liquid-phase droplets

**Fig 3:**
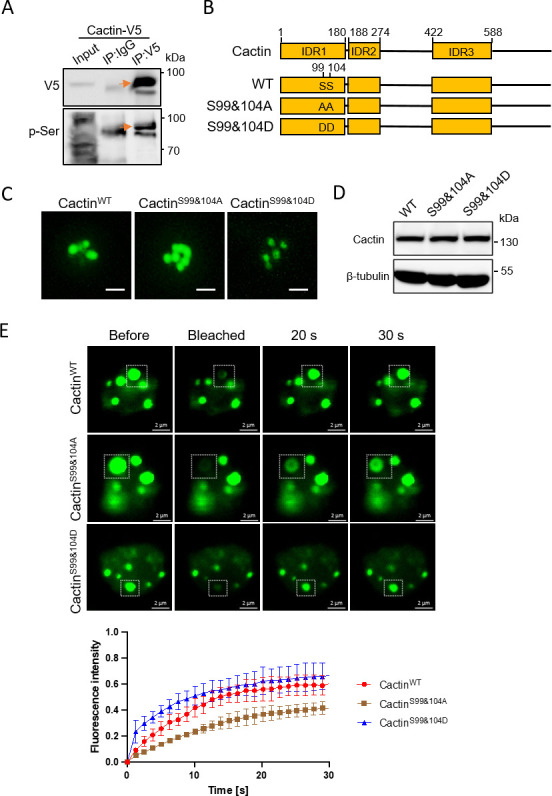
Phosphorylation of the IDR1 domain in Cactin regulates phase separation. (**A**) Phosphorylation of Cactin-V5 in S2 cells, detected using an anti-V5 antibody for Cactin-V5 enrichment and an anti-pSer antibody for detecting Cactin-V5 phosphorylation. The orange arrow indicates the phosphorylated form. Representative results from triplicate experiments are shown. (**B**) Schematic representation of Cactin phosphorylation mutants, highlighting the positions of two point mutations within Cactin variants (Cactin^S99&104A^ and Cactin^S99&104D^). (**C**) Confocal images showing the condensed state of Cactin^WT^-eGFP, Cactin^S99&104A^-eGFP, and Cactin^S99&104D^-eGFP in S2 cells (scale bars, 5 µm). (**D**) Western blotting analyses of Cactin^WT^-eGFP, Cactin^S99&104A^-eGFP, and Cactin^S99&104D^-eGFP in panel C. Representative results from triplicate experiments are shown. (**E**) FRAP measurement of Cactin^WT^-eGFP, Cactin^S99&104A^-eGFP, and Cactin^S99&104D^-eGFP in S2 cells (scale bar, 2 µm). Montages on the top show the processes of FRAP of droplets. The graph on the bottom shows the recovery of fluorescence intensity over time. Data correspond to the mean ± SD with *n* = 3.

Phosphorylation, a well-studied post-translational modification, has been recognized for its ability to modulate phase separation processes (49, 50). To explore the potential phosphorylation of the Cactin protein, we transiently expressed the fusion protein Cactin-V5 in S2 cells and conducted immunoprecipitation using an anti-V5 antibody. The immunoprecipitated Cactin protein was subjected to mass spectrometry analysis to identify phosphorylation modification sites (Table S1). This analysis revealed phosphorylation at two serine residues, specifically the 99th and 104th amino acids (^99^SPLVKS^104^), within the IDR1 domain of the Cactin protein. To validate the phosphorylation sites identified by mass spectrometry, the immunoprecipitated Cactin-V5 was probed using Western blotting with antibodies targeting phosphorylated serine (anti-pSer) and V5. The resulting findings revealed the abundance and phosphorylation status of Cactin (Fig. 3A), confirming that the Cactin protein undergoes phosphorylation in S2 cells.

We hypothesized that the phosphorylation status of the IDR1 domain of Cactin could affect its ability to undergo phase separation. To test this hypothesis, we used site-directed mutagenesis to replace the phosphorylated serine residues in IDR1 with either alanine (Cactin^S99&104A^-eGFP) or aspartic acid residues (Cactin^S99&104D^-eGFP), which mimicked the non-phosphorylated and hyperphosphorylated states of Cactin^WT^-eGFP, respectively (Fig. 3B). Confocal microscopy showed that Cactin^S99&104A^-eGFP tended to form larger aggregates than Cactin^WT^-eGFP, while Cactin^S99&104D^-eGFP formed smaller diffuse droplets (Fig. 3C). Importantly, the abundance of Cactin^WT^-eGFP, Cactin^S99&104A^-eGFP, and Cactin^S99&104D^-eGFP appeared to be comparable (Fig. 3D). Fluorescence recovery rate analysis using FRAP revealed that Cactin^S99&104D^-eGFP exhibited faster fluidity than Cactin^WT^-eGFP, while Cactin^S99&104A^-eGFP showed slower fluidity (Fig. 3E). Taken together, these results suggest that Cactin undergoes a transition from stable aggregates to more dynamic liquid-phase droplets upon phosphorylation of the IDR1 domain.

### Phosphoglycerate kinase regulates Cactin phosphorylation and phase separation

To investigate the kinases responsible for regulating Cactin phosphorylation, we performed co-immunoprecipitation (Co-IP) to isolate the Cactin–protein complex from DCV-infected S2 cells. We observed that a specific 50-kDa band was enriched in cells overexpressing Cactin-V5, regardless of DCV infection, compared to the control. Using mass spectrometry, we identified PGK as a protein interacting with Cactin (Table S2). Because the human homolog of PGK, PGK1, can act as a protein kinase to phosphorylate and activate PDHK1 (51), we hypothesized that PGK may regulate Cactin phosphorylation.

**Fig 4:**
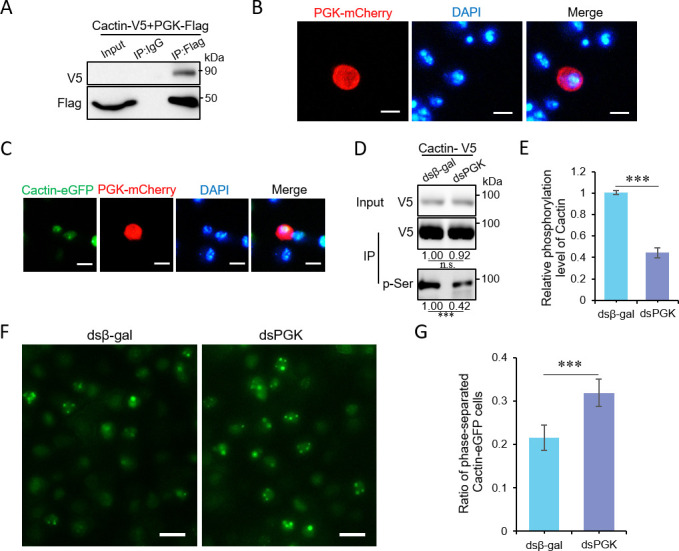
PGK-mediated phosphorylation regulates the phase separation of Cactin. (**A**) Immunoprecipitation performed in S2 cells transfected with pMT-PGK-Flag and pMT-Cactin-V5 plasmids. Representative results from triplicate experiments are shown. (**B**) *Drosophila* S2 cells transfected with pMT-PGK-mCherry plasmids, stained with DAPI to locate the nucleus. Confocal images illustrating the location of PGK-mCherry in S2 cells (scale bar, 5 µm). (**C**) *Drosophila* S2 cells transfected with pMT-Cactin-eGFP and pMT-PGK-mCherry plasmids, stained with DAPI to locate the nucleus. Confocal images showing the colocalization of Cactin-eGFP and PGK-mCherry in S2 cells (scale bar, 5 µm). (**D–E**) Cells transfected with pMT-Cactin-V5 plasmids 24 h earlier, treated with dsRNAs against control (β-gal) or PGK, followed by 48 h for immunoprecipitation. Anti-V5 antibody was used to detect Cactin-V5 enrichment, and phosphorylation of Cactin-V5 was detected with an anti-pSer antibody (**D**). Phosphorylated Cactin band density was normalized to total Cactin band density and compared to the control (**E**). Error bars represent the SD of three replicates. Representative results from triplicate experiments are shown in D. Data correspond to the mean ± SD with *n* = 3. ∗∗∗*P* < 0.001 (Student’s *t*-test). (**F–G**) Confocal images showing phase-separated cells in stable Cactin-eGFP-expressing cells pretreated 48 h earlier with dsRNAs against control (β-gal) or PGK, followed by a 12-h period of induced expression (**F**) (scale bar, 20 µm). The ratio of Cactin-eGFP cells undergoing phase separation was calculated (**G**). A total of eight fields were quantified, with an average of about 45 cells in each field. Data correspond to the mean ± SD with *n* = 8. ∗∗∗*P* < 0.001 (Student’s *t-*test).

To test this hypothesis, we first confirmed the interaction between Cactin and PGK in S2 cells transfected with pMT-Cactin-V5 and pMT-PGK-Flag plasmids by performing co-immunoprecipitation. Western blot analysis revealed that Cactin indeed interacted with PGK in S2 cells (Fig. 4A). Subsequently, we determined the subcellular localization of PGK. Upon transient expression of PGK-mCherry in S2 cells, we observed the distribution of PGK in both the nucleus and the cytoplasm (Fig. 4B). Next, we transiently expressed PGK-mCherry in cell lines stably expressing Cactin-eGFP, which led to the observation of Cactin droplet formation within the nucleus via phase separation. Notably, these Cactin droplets showed colocalization with PGK (Fig. 4C).

Furthermore, we investigated the effect of PGK knockdown on Cactin phosphorylation levels and phase separation. Western blot analysis showed that the protein content of Cactin-V5 remained constant, while the phosphorylation level of Cactin-V5 decreased significantly in cells with PGK knockdown (Fig. 4D and E), indicating that PGK regulates the phosphorylation level of Cactin. To further explore this, we employed a stable cell line expressing Cactin-eGFP to assess the effect of PGK on the phase separation state following Cactin phosphorylation. Following PGK knockdown, a higher proportion of cells exhibited Cactin aggregates at 12 h of induced expression compared to control cells (Fig. 4F and G). These results suggest that PGK-mediated phosphorylation modulates the phase separation process of Cactin.

### Phosphorylation enhances the antiviral ability of Cactin by promoting liquid-phase droplet formation

**Fig 5:**
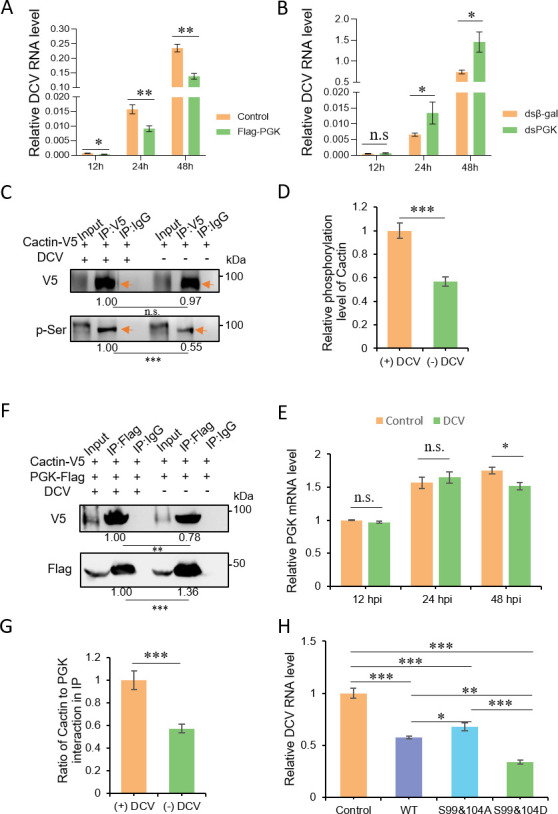
Cactin phase separation regulates DCV replication. (**A**) S2 cells were transfected with pMT-PGK-Flag or pMT-eGFP (control) plasmids for 48 h and then infected with DCV, and reverse transcription quantitative PCR (RT-qPCR) analysis of DCV RNA levels at 12, 24, and 48 hpi, shown values relative to those of *rp49*. Data correspond to mean ± SD with *n* = 3. ∗*P* < 0.05, ∗∗*P* < 0.01 (Student’s *t*-test). (**B**) S2 cells were pretreated with dsRNAs against a control (β-gal) or PGK for 48 h and then infected with DCV, and RT-qPCR analysis of DCV RNA levels at 12, 24, and 48 hpi, shown values relative to those of *rp49*. Data correspond to mean ± SD with *n* = 3. ∗*P* < 0.05, n.s., not significant (Student’s *t*-test). (**C–D**) Immunoprecipitation in S2 cells transfected with pMT-Cactin-V5 plasmids, uninfected or infected, with DCV (infection initiated 48 h after transfection). Anti-V5 antibody detected Cactin-V5 enrichment, and phosphorylation of Cactin-V5 was detected with an anti-pSer antibody (**C**). Phosphorylated Cactin band density was normalized to the total Cactin band density and compared to the control (**D**). Representatives from triplicate experiments are shown (**C**). Data correspond to mean ± SD with *n* = 3. ∗∗∗*P* < 0.001 (Student’s *t*-test). (**E**) Reverse transcription quantitative PCR (RT-qPCR) analysis of PGK gene expression levels at 12, 24, and 48 h in DCV-infected S2 cells, shown values relative to those of *rp49*. Data correspond to mean ± SD with *n* = 3. ∗*P* < 0.05; n.s., not significant (Student’s *t*-test). (**F–G**) Immunoprecipitation in S2 cells transfected with pMT-Cactin-V5 or pMT-PGK-Flag plasmids, uninfected or infected, with DCV (infection initiated 48 h after transfection) (**F**). Quantification of Cactin and PGK interactions under conditions of DCV infection and non-infection (**G**). Representatives from triplicate experiments are shown in F. Data correspond to mean ± SD with *n* = 3. ∗∗∗*P* < 0.001 (Student’s *t*-test). (**H**) S2 cells were transfected with pMT-Cactin^WT^-eGFP, pMT-Cactin^S99&104A^-eGFP, pMT-Cactin^S99&104D^-eGFP, and pMT-eGFP plasmids (negative control) for 48 h and then infected with DCV, and viral RNA quantification with RT-qPCR at 48 hpi, shown values relative to those of *rp49*. Data correspond to mean ± SD with *n* = 3. ∗*P* < 0.05; ∗∗*P* < 0.01; ∗∗∗*P* < 0.001 (Student’s *t*-test).

In conjunction with the transcription factor Deaf1, Cactin activates a non-canonical immune signaling pathway that regulates the expression of immune genes and limits the replication of DCV (40). Therefore, we investigated whether PGK-mediated phosphorylation could enhance the antiviral ability of Cactin by transforming it into a more dynamic liquid-phase droplet. To test this hypothesis, we overexpressed PGK in S2 cells and infected them with DCV. Reverse transcription quantitative PCR (RT-qPCR) analysis at 12, 24, and 48 h post-infection (hpi) revealed that PGK overexpression significantly inhibited DCV replication compared to control cells (Fig. 5A). Confirmation of this inhibition came from monitoring lower viral titers in PGK-overexpressing cells compared to control cells at 48 hpi (Fig. S1A). In contrast, PGK knockdown significantly increased DCV replication, leading to elevated viral RNAs and titers in knockdown cells (Fig. 5B; Fig. S1B). These results suggest that PGK can affect the replication of DCV and that PGK-mediated phosphorylation may enhance the antiviral ability of Cactin.

After transiently expressing Cactin-V5 in S2 cells and infecting them with DCV, we investigated the impact of DCV on Cactin phosphorylation. We observed an increase in Cactin phosphorylation in S2 cells infected with DCV compared to the uninfected control (Fig. 5C and D). Because PGK is responsible for Cactin phosphorylation, we examined the expression levels of PGK mRNA following DCV infection of S2 cells. RT-qPCR analysis revealed that there was no increase in PGK mRNA expression 12, 24, and 48 hpi after DCV infection (MOI = 1) (Fig. 5E). However, the interaction between Cactin and PGK was significantly enhanced after DCV infection (Fig. 5F and G). These findings suggest that DCV infection does not directly increase the mRNA levels of PGK but rather enhances the interaction between PGK and Cactin through an unknown mechanism. Therefore, PGK-mediated phosphorylation regulates the Cactin phase separation process upon DCV infection.

As unphosphorylated Cactin forms highly concentrated, stable aggregates through LLPS, which gradually transition into more dynamic liquid droplets upon phosphorylation, we investigated whether there were differences in the antiviral immunity of Cactin^S99&104D^, Cactin^WT^, and Cactin^S99&104A^. Our results demonstrated a shift from highly concentrated, stable oligomers to more dynamic liquid-phase droplets, enhancing the antiviral capability of Cactin (Fig. 5H; Fig. S2A).

To further investigate the impact of PGK-mediated phosphorylation on Cactin phase separation and antiviral activity, we knocked down either β-gal (control) or PGK and then transfected cells with pMT-Cactin^WT^-eGFP, pMT-Cactin^S99&104A^-eGFP, and pMT-Cactin^S99&104D^-eGFP plasmids. After 12 h of induction and subsequent DCV infection, confocal microscopy revealed that, compared to the control group, Cactin^WT^-eGFP exhibited a higher propensity for droplet formation, while the two mutant variants did not show a significant change compared to Cactin^WT^-eGFP (Fig. S2B).

RT-qPCR analysis revealed that knocking down PGK resulted in a decrease in the antiviral capacity of both Cactin^WT^-eGFP and the two mutant variants. Furthermore, the antiviral capabilities of Cactin^WT^-eGFP and Cactin^S99&104A^-eGFP were found to be similar (see Fig. S2C and D). These findings indicate that Cactin protein phase separation plays a critical role in regulating the replication of DCV.

## DISCUSSION

Our previous study demonstrated that Cactin activates a novel non-canonical immune signaling pathway to regulate the expression of immune genes and limit the replication of DCV by synergizing with the transcription factor Deaf1 (40). In this study, we show that PGK phosphorylates the Cactin protein and regulates its phase separation process in DCV-infected S2 cells. This transformation from a relatively stable condensed phase to a more dynamic liquid-phase droplet enables quick interaction with other components in the environment and exerts antiviral ability, ultimately inhibiting the replication of DCV.

Cactin is highly conserved in eukaryotes (41) and includes an NLS, a CC domain, and a region with relatively low complexity that is rich in arginine and serine residues (42). Cactin was initially discovered as a protein that interacts with Cactus in *Drosophila*, hence earning its name “Cactin” (short for Cactus interactor) (41). Cactus serves as an inhibitor of the Toll signaling pathway by binding to the NF-κB transcription factor Dorsal, preventing Dorsal from translocating into the nucleus (41). Cactin has been observed to form nuclear speckles in various organisms, including *Arabidopsis* and humans, suggesting the conservation of this property (52–56). Our studies demonstrate that Cactin is capable of undergoing LLPS, resulting in the formation of spherical liquid droplets. While the outcomes are largely based on results from Cactin overexpression, considering the conservation of Cactin-phase separation, it is reasonable to speculate that phase separation may also occur in the native state of Cactin in *Drosophila*. Biomolecular condensates are often driven by LLPS with modular domains, or IDRs, a type of domain that provides weak multivalent intermolecular interactions (8, 13, 14). Our results demonstrated that the IDR1 domain of Cactin mainly regulates phase separation. Our findings revealed that Cactin-GFP droplets exhibit a high propensity for fusion and rapid recovery after bleaching, as assessed through FRAP. This observation indicated a dynamic exchange of proteins between the droplet and the surrounding soluble proteins.

Numerous investigations have explored the regulation of phase separation from the perspective of post-translational protein modifications. Among these, phosphorylation, a widely studied post-translational modification, has been shown to modulate the process of phase separation (49, 50). Phosphorylation is the covalent attachment of a phosphate group to the hydroxyl group of an amino acid, which is negatively charged, changing a polar, uncharged residue into a negatively charged amino acid (57), possibly facilitating certain charge–charge interactions, thereby driving complex aggregations (phase separation of oppositely charged polymers) (58). Alternatively, it can cause charge repulsion or steric hindrance, inhibiting phase separation (5). Our research revealed that the Cactin protein undergoes phosphorylation and modification within S2 cells. Specifically, we identified two serine residues (^99^SPLVKS^104^) within the IDR1 domain of the Cactin protein that undergoes phosphorylation. Phosphorylation of the IDR1 domain converts non-charged serine residues into negatively charged residues. FRAP analysis of fluorescence recovery rate revealed that Cactin^S99&104D^-eGFP exhibited faster mobility than Cactin^WT^-eGFP, while Cactin^S99&104A^-eGFP revealed slower mobility (Fig. 3E). Therefore, unphosphorylated Cactin forms highly concentrated and stable aggregates through LLPS, but after phosphorylation, it gradually transforms from stable aggregates to more dynamic liquid-phase droplets. This suggests that phosphorylation inhibits the internal interaction of the protein, making it easier to interact with other components in the environment and exert its function (49, 50). The FRAP and phase fusion results demonstrated that phase separation was a highly dynamic process, transitioning gradually from small to larger foci. Therefore, the coexistence of both diffuse distribution and foci formation by Cactin is reasonable under these dynamic conditions (Fig. 1).

In this study, we identified PGK protein as a potential interacting protein of Cactin. PGK is known to activate PDHK1 as a protein kinase (51). Through co-IP experiments, we confirmed that PGK can also act as a protein kinase to phosphorylate the Cactin protein and regulate its phase separation process. Knocking down PGK in S2 cells resulted in a reduction in Cactin protein phosphorylation levels and a shift from dispersed liquid phase droplets to a relatively stable condensed phase. Our findings show that phosphorylated Cactin exhibits enhanced antiviral ability. Moreover, exogenously expressing or knocking down the PGK protein and infecting the DCV virus resulted in altered replication rates of the virus, suggesting that PGK can regulate the replication of DCV. These results highlight the role of PGK as an upstream regulatory component of Cactin protein antiviral ability by phosphorylating Cactin and regulating its phase separation process.

## MATERIALS AND METHODS

### Virus preparation and titration

DCV virions were purified following a previously published protocol with minor modifications (59). Briefly, S2 cells were inoculated with crude virions and collected after three days. The cells were lysed with 0.5% Nonidet P-40 and subjected to three freeze/thaw cycles. After digestion with RNase A, the sample was placed on a 30% sucrose cushion containing 0.05 M HEPES (pH 7.0), 5 mM CaCl_2_, 0.1% β-mercaptoethanol, and 0.1% bovine serum albumin, and then ultracentrifuged at 25,000 rpm for 2 h. The pellet was resuspended in 500 µL HEPES buffer, and the insoluble material was removed by centrifugation at 14,000 rpm at 4°C. The virion suspensions were aliquoted and stored at −80°C for future study. Virus stocks were titered by end-point dilution, and S2 cells, 2.5 × 10^5^ per well in a 96-well plate, were inoculated with a 10-fold dilution of the virus stocks. The cytopathic effect was monitored visually over 14 days. Titers were calculated as tissue culture infectious dose (TCID) 50/mL according to the Spearman–Karber method.

The DCV-infected cells were harvested at the designated time point through centrifugation. Total RNA was extracted from S2 cells using TRIzol (Invitrogen) and treated with DNase I (Thermo Fisher Scientific) to eliminate DNA contamination before detecting intracellular viral RNA levels through RT-qPCR. For the viral titer assay, the cell culture medium containing DCV-infected cells underwent three freeze/thaw cycles directly, followed by centrifugation at 10,000 rpm for 5 min. The resulting suspensions were titrated through end-point dilution on S2 cells to detect both intracellular and secreted virus particles.

### Cell culture and gene knockdown by RNAi

*Drosophila* S2 cells were cultured at 25°C in Schneider’s Insect Medium (Sigma) supplemented with 10% heat-inactivated (30 min at 56°C in a water bath) fetal bovine serum (GIBCO), 5 mM sodium bicarbonate (Sigma), 5 mM calcium chloride (Sigma), 100 U/mL penicillin, and 100 µg/mL streptomycin (HyClone, USA). First, cDNAs were prepared from S2 cells, and DNA templates were obtained by PCR using sets of primers containing the T7 polymerase recognition sequence at their 5′ end. Then, the dsRNAs of each gene were synthesized using a T7 transcription kit (Toyobo, Japan) and visualized using agarose gel electrophoresis. Approximately 1.5 × 10^6^ S2 cells were bathed in 400 µL of serum-free medium containing 7 µg of dsRNA per well of a 12-well plate at 25°C for 30 min. The soaked cells were then supplemented with 400 µL complete medium containing 20% FBS and incubated for 2 days at 25°C. Silenced cells were infected with DCV (MOI = 1) and assayed at the indicated post-infection time.

### Expression plasmids of *Drosophila* proteins

The pMT/V5-His A vector was used to express all proteins in S2 cells. The CDSs of *Drosophila* proteins were amplified by RT-PCR using gene-specific primers from the total RNA of S2 cells, and these cDNAs were cloned into the pMT/V5-His A vector. All plasmids were confirmed using Sanger sequencing.

### Transfection of plasmids into S2 cells

For DNA transfection, *Drosophila* S2 cells were plated in 12-well plates and grown for 4–6 h to reach 80% confluence (approximately 2 × 10^6^ cells per well). Then, 1 µg of plasmid DNA was transfected into the cells using Lipofectamine 3000 Transfection Reagent (Invitrogen) according to the manufacturer’s protocol. After culture at 25°C for 24 h, the cells were either stimulated or not with 25 µM CuSO_4_ for an additional 24 h. Transfected cells were harvested and directly processed to extract total RNA or protein.

### Western blot analysis

*Drosophila* S2 cells were harvested and washed twice with ice-cold PBS buffer. Then, the cells were lysed in 80 µL of cold lysis buffer [20 mM Tris-HCl pH 7.0, 50 mM NaCl, 0.5 mM EDTA, 0.5% NP40, 0.5% sodium deoxycholate, 1mM PMSF (Sangon Biotech), and protease inhibitor cocktail (Roche)] and kept on ice for 60 min. The cell extracts were spun at 12,000rpm for 10min at 4°C, and the supernatants were collected as the total protein extracts. The protein extracts were run on a 10% SDS‒PAGE gel and transferred to a PVDF membrane (Millipore). The membrane was blocked and then incubated with the indicated primary antibodies overnight at 4°C. After washing, the membranes were incubated withHorseradish peroxidase (HRP)-labeled goatanti-mouse/rabbit IgG secondary antibody (Beyotime, China) for1h. Immunoreactive bands were developed using BeoECL plus chemiluminescence substrate (Beyotime) and analyzed using LAS4000 (GEHealthcare). Quantifications of Western bands were performed using ImageJ.

### Reverse transcription quantitative PCR

Total RNA was extracted from S2 cells using TRIzol (Invitrogen) and treated with DNase I (Thermo Fisher Scientific) to remove DNA contamination. Reverse transcription was performed with random primers using the RevertAid First Strand cDNA Synthesis Kit (Fermentas, USA) according to the manufacturer’s instructions. RT-qPCR was conducted on a LightCycler 96 System (Roche) using the FastStart Essential DNA Green Master mix. All reactions were performed in triplicate, and the expression level for target genes versus the control *rp49* was calculated using the 2^-ΔΔCt^ method (60).

### Immunoprecipitation

S2 cells were transfected using Cactin-V5 expressing plasmids and then infected with DCV (MOI=1) for 48 h, if necessary. For IP, cells (5×10^7^) were collected and suspended in lysis buffer (2 mM EDTA, 50 mM Tris-HCl pH 7.4, 150 mM NaCl, 1% NP-40, 20% glycerol) supplemented withPhenylmethanesulfonyl fluoride (PMSF) (Sangon Biotech) on ice for 30 min. After centrifugation at 13,000 rpm for 10 min at 4°C, the lysates were incubated for 4 h with the anti-V5 tag antibody (Invitrogen) at 4°C, followed by further incubation with Protein A/G Agarose for1h. The beads were washed five times with lysis buffer. The proteins were eluted by boiling the beads for 5 min in 1 × SDS loading buffer and analyzed by Western blot with the anti-V5 tag (Invitrogen) or anti-p-Ser/phosphoserine (Santa Cruz).

### Co-immunoprecipitation

S2 cells were co-transfected with Cactin-V5 and PGK-Flag expressing plasmids, respectively, and then infected with DCV (MOI = 1) for 48 h if necessary. For Co-IP, cells (5 × 10^7^) were collected and suspended in lysis buffer (2 mM EDTA, 50 mM Tris-HCl pH 7.4, 150 mM NaCl, 1% NP-40, 20% glycerol) supplemented with PMSF (Sangon Biotech) on ice for 30 min. After centrifuging at 13,000 rpm for 10 min at 4°C, the lysates were incubated for 4 h with the anti-V5 tag antibody (Invitrogen) at 4°C, followed by further incubation with Protein A/G Agarose for 1 h. The beads were washed five times with lysis buffer. The proteins were eluted by boiling the beads for 5 min in 1× SDS loading buffer and analyzed by western blotting with the anti-V5 tag (Invitrogen) and anti-Flag tag (Sigma).

### Generation of stable cell lines

In a 12-well plate,1 mL of culture medium and 1.5 × 10^6^ S2 cells per well were added. The plate was incubated at 25°C for 3–6 h. Plasmid transfection was performed by mixing 50 µL of opti-MEMTM medium with 3 µL of Lipofectamine 3000 Transfection Reagent (Invitrogen) to prepare Solution A, and 50 µL of opti-MEMTM medium was mixed with 1 µg of the plasmid to prepare Solution B. Solution A and Solution B were combined, the mixture was left to stand for 20 min, and the S2 cells were added. The plate was incubated at 25°C for 48 h. The cells were collected through centrifugation at1000 rpm for 5 min,the supernatant was removed, and the cells were resuspended in a selection medium containing 5 µg/mL puromycin. The cells were seeded at a 1:1:2 ratio in two 12-well plates and one 6-well plate. The plates were incubated at 25°C for 4–6 days. The selection medium was replaced with puromycin-free culture medium when approximately 20% of unaffected cells remained in each well. The cells were incubated at 25°C until they fully occupied the well. Enrichment was induced by adding CuSO_4_, and the enrichment effect was observed. After two to three rounds of general drug screening, the desired cells were enriched.

### Fluorescence recovery after photobleaching

S2 cells were transfected with Cactin^WT^-eGFP, Cactin^S99&104A^-eGFP, or Cactin^S99&104D^-eGFP plasmids. Twenty-four hours post-transfection, live-cell confocal microscopy was used to perform FRAP on punctate regions by drawing a region of interest representing an equivalent area in the nucleus with the Cactin protein only. Imaging was completed on a ZEISS LSM880 confocal microscope using a Plan Fluor 40× oil DIC objective. For photobleaching, a laser wavelength of 488  nm with a laser power setting of 100% was utilized. Each experiment used 5  s of prebleaching acquisition, with 2–3 min of recovery.

### Live-cell imaging

S2 cells were seeded in 12-well glass-bottom culture plates and transfected with the indicated plasmids. Twenty-four hours after transfection, live-cell imaging was conducted using a ZEISS LSM880 confocal microscope with a 40× objective.

### Quantification and statistical analysis

Data were analyzed using an unpaired two-tailed Student’s *t*-test using GraphPad Prism software. Statistical significance is defined as ∗ *P* < 0.05, ∗∗ *P* < 0.01, and ∗∗∗ *P* < 0.001. Error bars represent SD in triplicate experiments.

## SUPPLEMENTAL MATERIAL

Supplemental Figures mbio.01378-23-s0001.pdf Fig. S1 and S2.

Table S1 mbio.01378-23-s0002.xlsx Potential phosphorylation modification sites of Cactin identified using MS.

Table S2 mbio.01378-23-s0003.xlsx Potential interacting proteins with Cactin identified using MS.

ASM does not own the copyrights to Supplemental Material that may be linked to, or accessed through, an article. The authors have granted ASM a non-exclusive, world-wide license to publish the Supplemental Material files. Please contact the corresponding author directly for reuse.
