# Supplementary material for: Regulation of phase separation and antiviral activity of Cactin by glycolytic enzyme PGK via phosphorylation in Drosophila
Source: mBio. 2024 Mar 6;15(4):e01378-23. doi: 10.1128/mbio.01378-23 (PMC11005415; doi:10.1128/mbio.01378-23)
Supplement: Supplemental Figures — Fig. S1 and S2. [file mbio.01378-23-s0001.pdf]

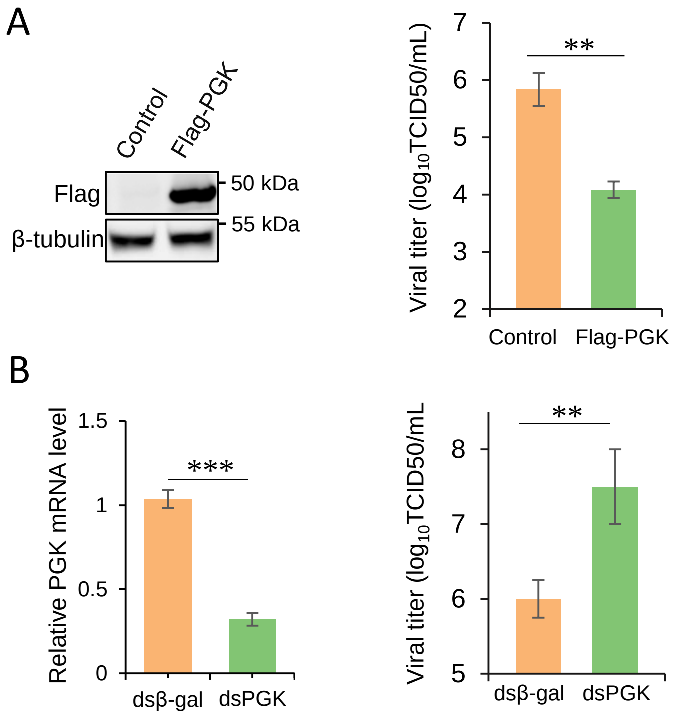

Fig. S1. PGK inhibit DCV replication in S2 cells.

A. S2 cells were transfected with pMT-PGK-Flag or pMT-eGFP (control) plasmids for 48 h, and then infected with DCV (MOI=1). PGK protein was detected by anti-Flag antibody at 48 h (left), and DCV viral titers were determined at 48 hpi (right). Representatives from triplicate experiments are shown, where β-tubulin was used as the control (left). Data correspond to mean ± SD with n = 3. $**p < 0.01$ (Student's t-test).

B. S2 cells were pretreated with dsRNAs against a control (β-gal) or PGK for 48 h, and then infected with DCV (MOI=1). At 48 h, PGK mRNA levels were determined by RT-qPCR relative to controls, shown as relative to the values of *rp49* (left). DCV viral titers were determined at 48 hpi (right). Data correspond to mean ± SD with n = 3. $**p < 0.01$, $***p < 0.001$ (Student's t-test).

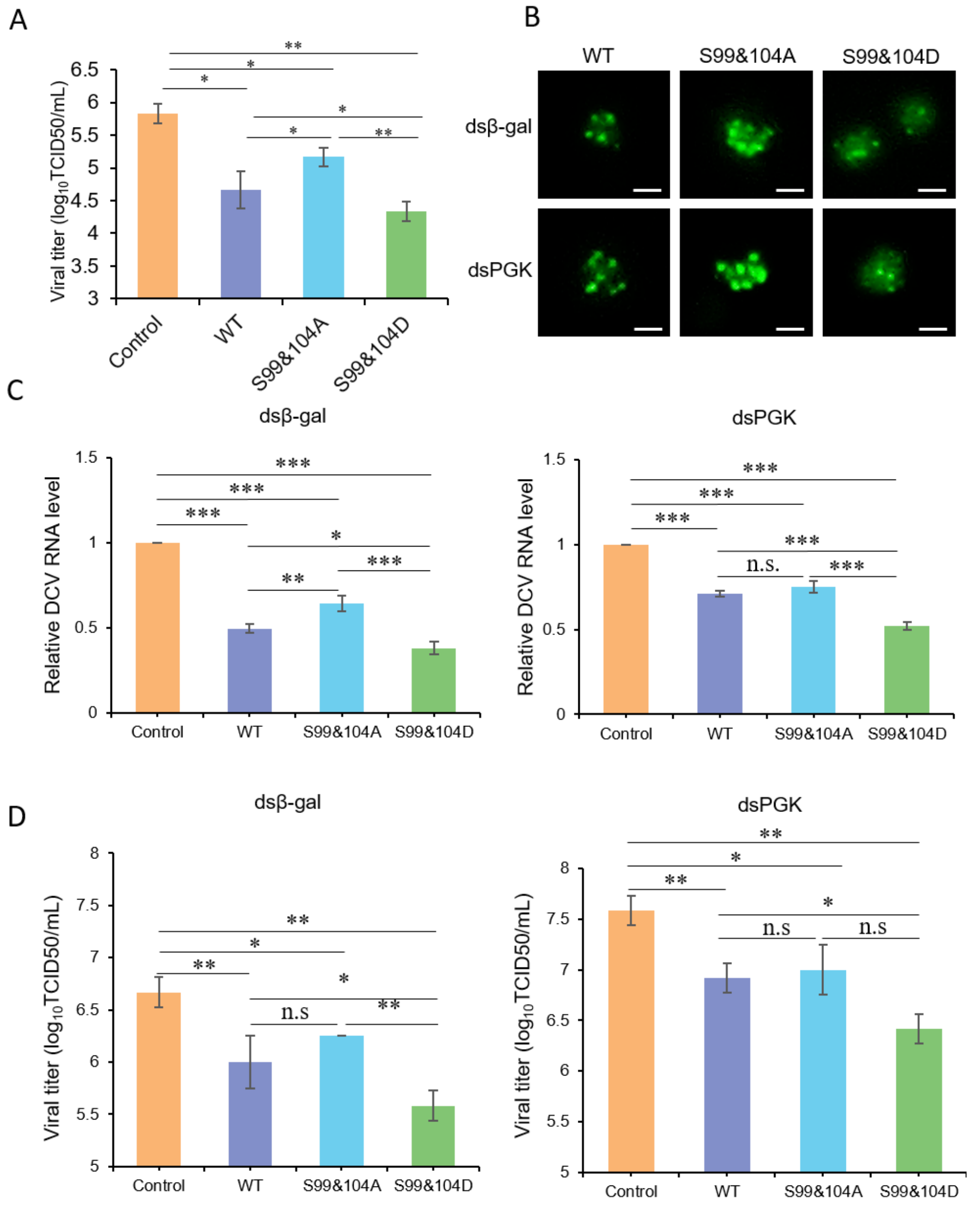

Fig. S2. PGK protein regulates Cactin phase separation and antiviral activity through phosphorylation of serine residues at positions 99 and 104

A. S2 cells were transfected with pMT-Cactin^{WT}-eGFP, pMT-Cactin$^{S99\&104A}$-eGFP, pMT-Cactin$^{S99\&104D}$-eGFP and pMT-eGFP plasmids (negative control) for 48 h, and then infected with DCV(MOI=1). DCV viral titers were determined at 48 hpi. Data correspond to mean ± SD with n = 3. $*p < 0.05$; $**p < 0.01$ (Student's t test).

B. Confocal images showing the condensed state of S2 cells 24 h after pre-treatment with dsRNA targeting the control (β-gal) or PGK, followed by transfection of pMT-Cactin^{WT}-eGFP, pMT-Cactin$^{S99\&104A}$-eGFP, pMT-Cactin$^{S99\&104D}$-eGFP plasmids (scale bar, 5 μm).

C. S2 cells were pretreated with dsRNA against a control (ß-gal) or PGK and followed by transfection of pMT-Cactin^{WT}-eGFP, pMT-Cactin$^{S99\&104A}$-eGFP, pMT-Cactin$^{S99\&104D}$-eGFP, and pMT-eGFP plasmids (negative control). Subsequently, the cells were infected with DCV at 48 h after transfection, and the relative DCV RNA levels were detected by RT-qPCR at 48 hpi, shown relative to the values of *rp49*. Data correspond to mean ± SD with n = 3. $*p < 0.05$; $**p < 0.01$; $***p < 0.001$; n.s., not significant (Student's t test).

D. S2 cells were pretreated with dsRNA targeting the control (β-gal) or PGK, followed by transfection of pMT-Cactin^{WT}-eGFP, pMT-Cactin$^{S99\&104A}$-eGFP, pMT-Cactin$^{S99\&104D}$-eGFP, and pMT-eGFP plasmids (negative control). Subsequently, the cells were infected with DCV at 48 h after transfection, and the viral titers were determined at 48 hpi. Data correspond to mean ± SD with n = 3. $*p < 0.05$; $**p < 0.01$; n.s., not significant (Student's t test).
